# Robust Regression Techniques for Multiple Method Comparison and Transformation

**DOI:** 10.1002/bimj.202400027

**Published:** 2024-07-13

**Authors:** Florian Dufey

**Affiliations:** ^1^ Roche Diagnostics GmbH Assay Development & System Integration (DSRIBF) Penzberg Germany

**Keywords:** Bland–Altman plots, clinical statistics, method comparison, Passing–Bablok regression, regression

## Abstract

A generalization of Passing–Bablok regression is proposed for comparing multiple measurement methods simultaneously. Possible applications include assay migration studies or interlaboratory trials. When comparing only two methods, the method boils down to the usual Passing–Bablok estimator. It is close in spirit to reduced major axis regression, which is, however, not robust. To obtain a robust estimator, the major axis is replaced by the (hyper‐)spherical median axis. This technique has been applied to compare SARS‐CoV‐2 serological tests, bilirubin in neonates, and an in vitro diagnostic test using different instruments, sample preparations, and reagent lots. In addition, plots similar to the well‐known Bland–Altman plots have been developed to represent the variance structure.

**Abbreviations**
iid: identically independently distributed
MAR: major axis regression
MC: method comparison
mPBR, mMAR, mRMR: multivariate PBR, MAR, and RMR, respectively,
OLS: ordinary least squares regression
PBR: Passing–Bablok regression
RMR: reduced major axis regression

## 1 Introduction

In clinical chemistry, the comparison of different measurement procedures for the same analyte is a recurring task. In this context, the measurement procedures are often referred to as “methods” and corresponding studies are known as “method comparison (MC) studies” (Altman and Bland 1983; Bland and Altman 1986). Typically, a set of patient samples is measured using both measurement procedures that are being compared, and some variant of linear regression is performed. Since both methods usually produce results with a comparable measurement error, the preferred analytical tools are regression methods commonly referred to as “errors in variables” models or “regression with measurement error” (Cheng and Van Ness 1999).

Various review articles (Bolfarine et al. 2020; Francq and Govaerts 2014; Linnet 1990; 1993) and guidelines (Clinical and Laboratory Standards Institute 2013; Hojvat and Kondratovic 2013) recommend specific regression methodologies for these studies. The recommended approaches are either variants of major axis regression (MAR), also known as Deming or orthogonal regression, or reduced major axis regression (RMR), also known as least product regression (Adcock 1878; Kummell 1879). Alternatively, if a more robust regression method is required to handle outlying measurements or nonlinear deviations near the boundaries, Passing–Bablok regression (PBR) is recommended (Bablok et al. 1988; Dufey 2020; Passing and Bablok 1983). In PBR, the slope estimator is obtained as a shifted median of all pairwise slopes.

While there are multidimensional extensions of MAR and RMR (Bolfarine et al. 2020; Feldmann et al. 1981) (mMAR and mRMR, respectively), which allow for the comparison of multiple measurement methods at the same time, there are currently no extensions of the PBR to the multiple method comparison case. The aim of this article is to extend the robust PBR methodology to cases where measurements obtained from more than two methods need to be compared. Additionally, the slope estimators to be obtained are required to be compatible in the sense that if ${\hat{\beta }}_{12}$ is the slope estimate from comparing method 1 and method 2, and ${\hat{\beta }}_{23}$ is the slope estimate between method 2 and method 3, then we require that the estimator of the slope between method 1 and method 3, denoted as ${\hat{\beta }}_{13}$, fulfills the relationship ${\hat{\beta }}_{13}={\hat{\beta }}_{12}{\hat{\beta }}_{23}$. It is worth noting that while the MAR and RMR estimates are compatible in this sense, the pairwise PBR estimates are not.

To address this issue, we propose a multidimensional Passing–Bablok regression (mPBR) method, which boils down to the ordinary PBR in two dimensions and provides compatible and robust estimates in more than two dimensions. In mPBR, we utilize the (hyper‐)spherical median axis instead of the major axis used in mRMR. The (hyper‐)spherical median axis is a concept derived from directional statistics and is defined as an axis with minimal angular distance to all points after projecting the points and the median axis onto a (hyper‐)sphere (Fisher, Lunn, and Davies 1993; Mardia, Jupp, and Mardia 2000).

We will apply this method to the comparison of SARS‐CoV‐2 serological tests (Ferrari et al. 2021), plasma bilirubin tests in neonates, and a clinical test that involves different sample preparation methods, instrument platforms, and reagent lots. In the latter example, we will demonstrate the utility of mPBR in analyzing differences between subgroups.

## 2 Mathematical Derivation of the New Regression Method

Consider a sample panel of size n where individual samples are labeled by i∈{1,…,n} each of which is measured with N different methods whose results are arranged into an array with components $x_{i\mu }$ with μ∈{1,…,N}. The values $x_{i\mu }$ are assumed to follow the functional model:

(1) $$x_{i\mu }=\beta_{\mu }r_{i}+\alpha_{\mu }+\varepsilon_{i\mu },$$

where $\beta :=\{\beta_{\mu }\}$ is the slope vector and the scalar $r_{i}$ is proportional to the unknown true concentration of sample i. The values $r_{i}$ are assumed fixed constants, also called latent variables. $\alpha :=\{\alpha_{\mu }\}$ is the intercept vector, and $\varepsilon_{i}:=\left\{\varepsilon_{i\mu }\right\}$ is the vector of random errors. Note that not only α and β but also the $r_{i}$ related to concentration are parameters that need to be estimated. To make the parameters unique, one degree of freedom of β and α may be constrained at will, for example, $\beta_{1}=1$ and $\sum_{\mu }\alpha_{\mu }=0$. Furthermore, the parameters $\beta_{\mu }$ and their estimates ${\hat{\beta }}_{\mu }$ are assumed to be positive. The random errors are assumed independent for i≠j. With the variances of the $\varepsilon_{i\mu }$ being $\sigma_{i\mu }^{2}$, it is assumed that the $\varepsilon_{i\mu }/\sigma_{i\mu }$ with different μ but equal index i are identically independently distributed (iid). The variance ratios $\lambda_{\mu }=\sigma_{i\mu }^{2}/\sigma_{i1}^{2}$ are assumed to be independent of i. With the matrix $\Lambda :=\mathrm{diag}(\lambda_{\mu })$, the variance matrix is $\Xi_{i}=\Lambda \sigma_{i1}^{2}$. Both the $\sigma_{i}:=\sigma_{i1}$ and the distribution of the $\varepsilon_{i\mu }/\sigma_{i\mu }$ may vary with the true concentration $r_{i}$, that is, the errors may be heteroscedastic. This generalizes the assumptions made in MAR or PBR (Bablok et al. 1988) for the comparison of two methods.

While for MAR, these assumptions are sufficient, in mRMR and mPBR, it is furthermore assumed that $\lambda_{\mu }=\beta_{\mu }^{2}$. It can be shown (cf. Appendix A) that the robust analog to mRMR to be developed in Section 2.2 is only consistent if the errors are normally distributed.

A useful equivalent expression is

$$y_{i\mu }:=b_{\mu }x_{i\mu }=r_{i}+\varepsilon_{i\mu }^{\prime }+a_{\mu },$$

with the vector of inverse slopes $b:=\{1/\beta_{\mu }\}$, which may be interpreted as scaling factors, the scaled intercept parameters are $a_{\mu }:=b_{\mu }\alpha_{\mu }$ and $\varepsilon_{i\mu }^{\prime }:=b_{\mu }\varepsilon_{i\mu }$.

### 2.1 Derivation of mMAR and mRMR with Zero Intercept

First, let us consider the situation a=0, that is, when the expected regression line passes through the origin. The estimated slope/scaling vector $\hat{\beta }$ is obtained from the centered principal component analysis: The equations of mMAR, are obtained by minimizing the sum of squares

(2) $$\sum_{i}{\left(x_{i}-\beta r_{i}\right)}^{T}\Xi_{i}^{-1}\left(x_{i}-\beta r_{i}\right)$$

with respect to the $r_{i}$ and β. Here, $x^{T}$ signifies the transpose of x. This yields the estimates

$${\hat{r}}_{i}=\frac{{\hat{\beta }}^{T}\Lambda^{-1}x_{i}}{{\hat{\beta }}^{T}\Lambda^{-1}\hat{\beta }}$$

and subsequently an equation for $\hat{\beta }$:

(3) $$\sum_{i}\sigma_{i}^{-2}\left({\hat{\beta }}^{T}\Lambda^{-1}x_{i}\right)\left(I_{N}-\frac{\Lambda^{-1/2}\hat{\beta }{\hat{\beta }}^{T}\Lambda^{-1/2}}{{\hat{\beta }}^{T}\Lambda^{-1}\hat{\beta }}\right)\Lambda^{-1/2}x_{i}=0,$$

where $I_{N}$ is the N×N diagonal unit matrix.

This is just the well‐known equation of weighted mMAR with $s:=\Lambda^{-1/2}\hat{\beta }$ being the vector pointing in the direction of the major axis. Measurements with a large value of $r_{i}$ will have a high leverage; hence, the method is not robust against outliers.

The equation for the mRMR slope estimate $\hat{\beta }$ follows with the special constraint^1^ $\Lambda_{\mu \mu }={\hat{\beta }}_{\mu }^{2}$ in Equation (3), that is, $\Lambda^{-1/2}$ is a diagonal matrix $\Lambda^{-1/2}=\mathrm{diag}\left(\hat{b}\right)$ (we assume all $\hat{b}$ to be positive). Introducing the diagonal matrix $X_{i}=\mathrm{diag}\left(x_{i}\right)$, we have the identity

(4) $$\Lambda^{1/2}x_{i}=X_{i}\hat{b}.$$

Finally,

(5) $$\begin{array}{cc} \sum_{i}\sigma_{i}^{-2}\left(e^{T}\Lambda^{1/2}{\hat{x}}_{i}\right)\left(I_{N}-ee^{T}\right)\Lambda^{1/2}{\hat{x}}_{i} & =0\text{, or,} \end{array}$$

(6) $$\begin{array}{cc} \sum_{i}\sigma_{i}^{-2}\left(e^{T}X_{i}\hat{b}\right)\left(I_{N}-ee^{T}\right)X_{i}\hat{b} & =0. \end{array}$$

### 2.2 Derivation of mPBR with Zero Intercept

**FIGURE 1:**
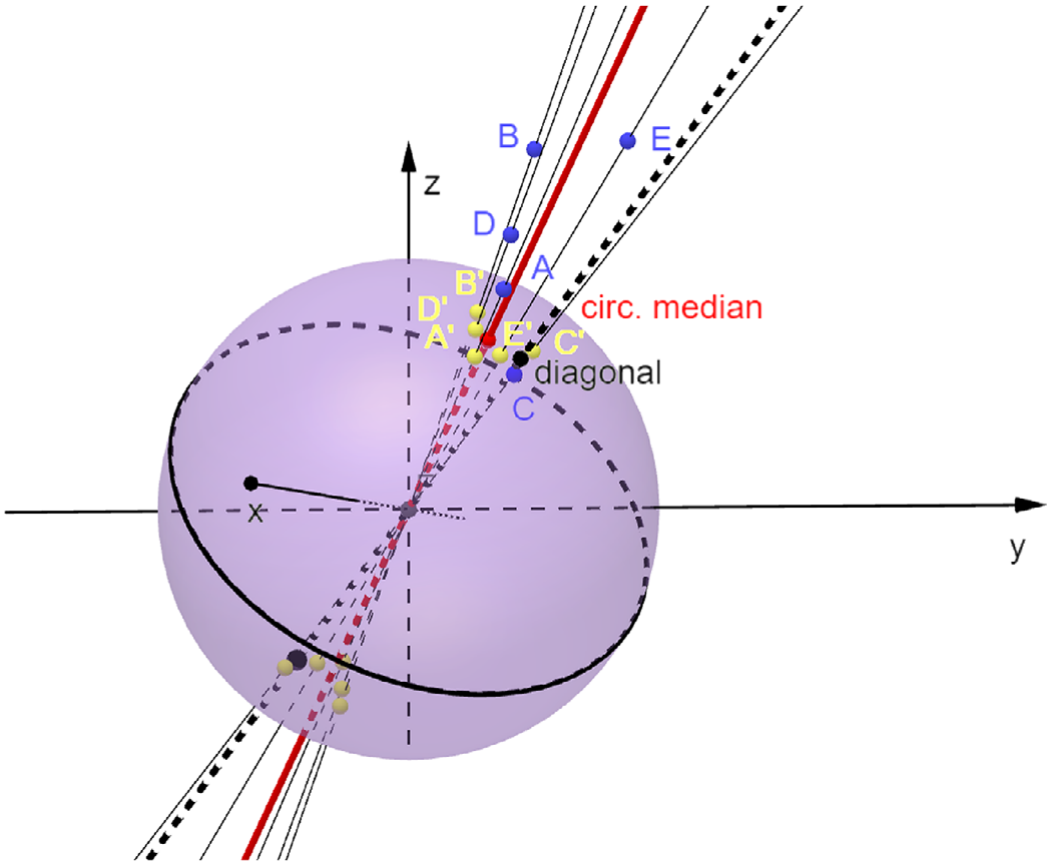
Spherical median axis (red) of 5 points (A–E, blue). The yellow points A'–E' are the projections of the points on the sphere. The red point corresponding to s designates the position of the spherical median. It has the smallest angular distance on the sphere to the points A'– E'. Only the points on the same hemisphere as s are taken into account. Λ=I was assumed for illustration.

The alternative mPBR algorithm utilizes ideas from finding the spherical median axis (Fisher, Lunn, and Davies 1993) (cf. Figure 1). In the spirit of most robust regression methods, all weights are assumed to equal $\sigma_{i}^{-2}=1$. Consider first the situation analogous to mMAR, where the matrix $\Lambda^{-1}$ is assumed to be diagonal, its elements are known and do not need to be estimated.

We consider straight lines passing through the origin and the scaled points $\Lambda^{-1/2}x_{i}$. With $\tilde{x}:=x/\left|x\right|$ signifying a vector of unit length with the same direction an x (not to be confused with the median), each of these lines will intersect the unit sphere at two antipodal points $\pm {\tilde{\Lambda^{-1/2}x}}_{i}=\pm \Lambda^{-1/2}x_{i}/\left|\Lambda^{-1/2}x_{i}\right|$. The circular median axis is then the line defined by the unit vector $\tilde{s}$ (remember $s=\Lambda^{-1/2}\hat{\beta }$) with the smallest sum of distances to all points ${\tilde{\Lambda^{-1/2}x}}_{i}$ on the same hemisphere. Mathematically, we seek to minimize the following expression:

(7) $$\underset{\tilde{s}}{\arg\min}\sum_{i}\arccos\left(\left|{\tilde{s}}^{T}{\tilde{\Lambda^{-1/2}x}}_{i}\right|\right).$$

For the case where Λ is a unit matrix, the situation is illustrated in Figure 1.

As long as none of the ${\tilde{\Lambda^{-1/2}x}}_{i}$ falls directly on the axis defined by $\tilde{s}$, the dependence on $\tilde{s}$ is analytical, and the minimum is attained if

(8) $$\sum_{i}\mathrm{sgn}\left({\tilde{s}}^{T}\Lambda^{-1/2}x_{i}\right)\frac{\left(I_{N}-\tilde{s}{\tilde{s}}^{T}\right)\Lambda^{-1/2}x_{i}}{\left|\left(I_{N}-\tilde{s}{\tilde{s}}^{T}\right)\Lambda^{-1/2}x_{i}\right|}=0.$$

While the corresponding regression estimate $\hat{\beta }\propto \Lambda^{1/2}\tilde{s}$ is robust against outlying measurements, it will be an unbiased estimate of β only if the errors follow a normal distribution (cf. Appendix A).

Therefore, the equivalent of the mRMR, obtained by setting $\Lambda_{\mu \mu }={\hat{\beta }}_{\mu }^{2}$ in Equation (8) is more attractive. With this constraint, the scaled errors $\varepsilon_{i\mu }^{\prime }$ are all from the same distribution for each i, so that the distribution of the $y_{i\mu }-a_{\mu }$ is symmetrical around the $r_{i}$. This is sufficient (cf. Appendix A) to guarantee the consistency of the mPBR estimator $\hat{b}$.

In this case, the spherical median $\tilde{s}$ of the $y_{i}$ after scaling is known to coincide with the space diagonal e, so Equation (8) becomes an equation for the estimated scaling vector $\hat{b}$:

(9) $$\underset{\tilde{s}}{\arg\min}\sum_{i}\arccos\left(\left|{\tilde{s}}^{T}\tilde{X_{i}\hat{b}}\right|\right)=e,$$

where $X_{i}$ was introduced in Equation (4).

**FIGURE 2:**
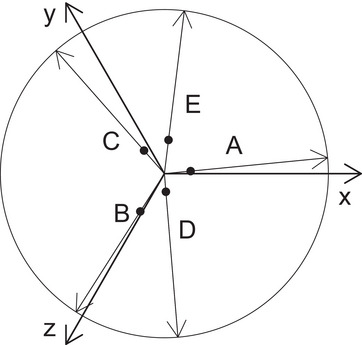
Visualization of the mPBR algorithm with zero intercepts: The points are scaled with the ${\hat{b}}_{\mu }$ until the unit vectors passing through the points A−D (filled dots) in the plane perpendicular to the space diagonal sum up to 0.

Hence, the equation for estimating $\hat{b}$ becomes

(10) $$\sum_{i}\mathrm{sgn}\left(x_{i}^{T}\hat{b}\right)\frac{PX_{i}\hat{b}}{\left|PX_{i}\hat{b}\right|}=0,$$

where $P=(I_{N}-ee^{T})$ is the projection matrix to the hyperplane perpendicular to e. Comparing Equations (5) and (10), we can see that the latter differs from the former by the missing weighting with $\sigma_{i}$, the replacement of $e^{T}PX_{i}\hat{b}$ by its sign, and the replacement of $PX_{i}\hat{b}$ by a unit vector pointing in the same direction. The influence of each term is bounded by ±1, making the estimate robust (Figure 2). 

The actual slope estimator of method ν versus method μ is ${\hat{\beta }}_{\mu \nu }={\hat{b}}_{\mu }/{\hat{b}}_{\nu }$.

These estimators fulfill the compatibility condition ${\hat{\beta }}_{\mu \nu }{\hat{\beta }}_{\nu \kappa }={\hat{\beta }}_{\mu \kappa }$.

By choosing ${\hat{b}}_{1}=1$, the scale of b and r is set to that of the first method. Then, the ${\hat{\beta }}_{\mu }=1/{\hat{b}}_{\mu }$ with μ>1 represent the N−1 slopes relative to that of the first method with ${\hat{\beta }}_{1}=1$.

### 2.3 Derivation of mPBR with Nonzero Intercept

If the intercept parameters are a priori not known to be 0, then, in the spirit of Theil–Sen and Passing–Bablok regression, the estimation of the scaling parameters b can be decoupled from the estimation of the intercept parameters a. To this end, we replace the $x_{i}$ in Equation (10) with the differences between the *x*‐values, denoted as $\Delta x_{J}:=x_{j}-x_{i}$ with i<j. The sum is extended to the corresponding index pairs {J}:={(i,j)}. As before (Equation 4), diagonal matrices $\Delta X_{J}$ are defined with $\Delta X_{J}=\mathrm{diag}\left(\Delta x_{J}\right)$. We get the final equation determining the slope vector $\hat{b}$ of the mPBR method:

(11) $$\sum_{J}\mathrm{sgn}\left(\Delta x_{J}^{T}\hat{b}\right)\frac{P\Delta X_{J}\hat{b}}{\left|P\Delta X_{J}\hat{b}\right|}=0.$$

In two dimensions, with $x_{i}^{\prime }:=x_{i1}$, $y_{i}^{\prime }:=x_{i2}$, ${\hat{\beta }}_{1}=1$ and ${\hat{\beta }}_{2}={\hat{\beta }}_{12}$, Equation (11) simplifies to

$$\sum_{J}\mathrm{sgn}\left(\Delta y_{J}^{\prime }+{\hat{\beta }}_{12}\Delta x_{J}^{\prime }\right)\mathrm{sgn}\left(\Delta y_{J}^{\prime }-{\hat{\beta }}_{12}\Delta x_{J}^{\prime }\right))=0.$$

This equation coincides with the defining equation of the pairwise PBR (Dufey 2020).

With the constraint $\sum_{\mu }a_{\mu }=e^{T}a=0$ made for the intercept vector,

$$E\left(\frac{x_{i}^{T}b}{N}\right)=r_{i}$$

we use

$${\hat{r}}_{i}=\frac{x_{i}^{T}\hat{b}}{N}$$

as an estimator for $r_{i}$. Then we obtain a robust estimator for the intercept vector ${\hat{a}}_{\mu }$ from the spatial median, denoted as SpMed (Weiszfeld 1937),

$${\hat{a}}_{\mu }=\mathrm{SpMed}\left(\{PX_{i}\hat{b}\}\right).$$

The spatial median SpMed is defined as the point with minimal distance from all the $x_{i}$:

$$\mathrm{SpMed}\left(\left\{x_{i}\right\}\right):=\underset{y}{\arg\min}\sum_{i}d\left(x_{i}-y\right),$$

where d(x) is the usual Euclidean norm of the vector x. This is equivalent to the equation:

$$\sum_{i}\frac{x_{i}-\hat{y}}{|x_{i}-\hat{y}|}=0,$$

as long as $\hat{y}$ does not equal any of the $x_{i}$ (adaptations for this situation are easily devised).

From the intercept parameter vector $\hat{a}$ the intercept $\alpha_{\mu \nu }$ for the graph of $x_{\nu }$ versus $x_{\mu }$, ${\hat{\alpha }}_{\mu \nu }$, can be calculated as

$${\hat{\alpha }}_{\mu \nu }:=\frac{{\hat{a}}_{\nu }-{\hat{a}}_{\mu }}{{\hat{b}}_{\nu }}={\hat{\alpha }}_{\nu }-{\hat{\alpha }}_{\mu }\frac{{\hat{b}}_{\mu }}{{\hat{b}}_{\nu }}.$$

### 2.4 Computation of the mPBR Estimates

A solution of Equation (11) (and similarly Equation 10) can be achieved through iterative reweighting (Beck and Sabach 2015; Weiszfeld 1937). To solve Equation (11), approximate values for the weights

$$w_{J}^{\left(n\right)}=\frac{\mathrm{sgn}(\Delta x_{J}^{T}{\hat{b}}^{\left(n\right)})}{\left|P\Delta X_{J}{\hat{b}}^{\left(n\right)}\right|}$$

can be calculated using the current estimator of the slopes ${\hat{b}}^{\left(n\right)}$ (a starting value could be all ${\hat{b}}_{\mu }^{\left(0\right)}=1)$. Subsequently, an improved estimator ${\hat{b}}_{\mu }^{\left(n+1\right)}$ can be derived by solving the linear equation system:

$$\sum_{J}w_{J}^{\left(n\right)}P\Delta X_{J}{\hat{b}}^{\left(n+1\right)}=0.$$

Similarly, the spatial median estimate of the intercept $\hat{a}$ can be computed. This can be done either after obtaining a converged estimate of the slope from

$$\sum_{i}v_{i}^{\left(n\right)}P\left({\hat{a}}^{\left(n+1\right)}-x_{i}\hat{b}\right)=0$$

with weights $v_{i}^{\left(n\right)}=\left|P\left({\hat{a}}^{\left(n\right)}-x_{i}\hat{b}\right)\right|$, or simultaneously by replacing $\hat{b}$ with ${\hat{b}}^{\left(n+1\right)}$ in these equations. It has been proven that slight extensions of the Weiszfeld algorithm (Beck and Sabach 2015) for the spatial median always converge. Eventually, it is possible to implement similar extensions for slope estimation. The simultaneous procedure offers the advantage of enabling to impute missing observations in the spirit of an expectation‐maximization algorithm (Dempster, Laird, and Rubin 1977).

### 2.5 Robustness of the Multivariate PBR Estimator

The examples presented in the next section showcase the potential of the new method. However, a formal proof of most of its statistical properties is not in the scope of this article. As consistency is a fundamental requirement of any estimator, proof of the stated conditions for consistency is given in Appendix A.

Another property, which has been claimed repeatedly in this article, is the robustness of the mPBR estimator. Several measures of robustness have been proposed (Cheng and Van Ness 1999); its quantification or even derivation of upper bounds is not easy, especially for multidimensional estimators (Davies and Gather 2005). Hence we will discuss a special case of the finite sample breakdown point (Davies and Gather 2005), when the correct values have vanishing error.

Considering first the mPBR with zero intercept, assume that of the n samples, $n_{O}$ are “outliers” while the remaining $n_{S}$ “correct” points obey the functional model Equation (1) with vanishing errors $\varepsilon_{i}$. Therefore, all correct points will be mapped to the same point S on the sphere. The outliers will have a maximal effect if all pull in the same direction, so we assume them to be projected on another point O which includes the angle $\varphi_{\mathrm{OS}}$ with S. The estimator E will fall on the great circle joining O and S between these two points, $0\leq \varphi_{\mathrm{ES}}\leq \varphi_{\mathrm{OS}}$, the sum of the angular distances to be minimized being

(12) $$\begin{array}{cc} \sum \varphi & =n_{S}\varphi_{\mathrm{ES}}+\left(n-n_{S}\right)\left(\varphi_{\mathrm{OS}}-\varphi_{\mathrm{ES}}\right), \end{array}$$

(13) $$\begin{array}{cc} & =\left(2n_{S}-n\right)\varphi_{\mathrm{ES}}+\left(n-n_{S}\right)\varphi_{\mathrm{OS}}. \end{array}$$

This is clearly minimal for $\varphi_{\mathrm{ES}}=0$ as long as $n_{O}/n<0.5$; the estimator is robust against outliers with a breakdown point (restricted to precise measurements) of 50%. The argument will remain qualitatively correct even if the measurement errors $\varepsilon_{i}$ are nonzero as long as the angular spread of the “correct” measurements is much smaller than $\varphi_{\mathrm{OS}}$.

For the mPBR estimator with nonzero intercept, a similar rational can be developed: There are $n_{\mathrm{OO}}=n_{O}\left(n_{O}-1\right)/2$ pairwise slopes between outliers, $n_{\mathrm{OS}}=n_{O}n_{S}$ slopes between outliers and correct measurements and $n_{\mathrm{SS}}=n_{S}\left(n_{S}-1\right)/2$ slopes between correct measurements. The $n_{\mathrm{SS}}$ precise measurement pairs are always projected on one point on the sphere, and it is possible for all other pairs to get projected on one single distinguished point O,^2^ which represents the worst‐case scenario. An analogous calculation replacing $n_{S}$ with $n_{\mathrm{SS}}$ and n with n(n−1)/2 in Equation (12) yields $n_{O}/n=1-\sqrt{2}/2$ for the breakdown point (of otherwise precise measurements) in the large sample limit. This coincides with the breakdown point of the Theil–Sen estimator.

## 3 Examples

### 3.1 Multidimensional Regression of SARS‐CoV‐2 Measurement Data

**FIGURE 3:**
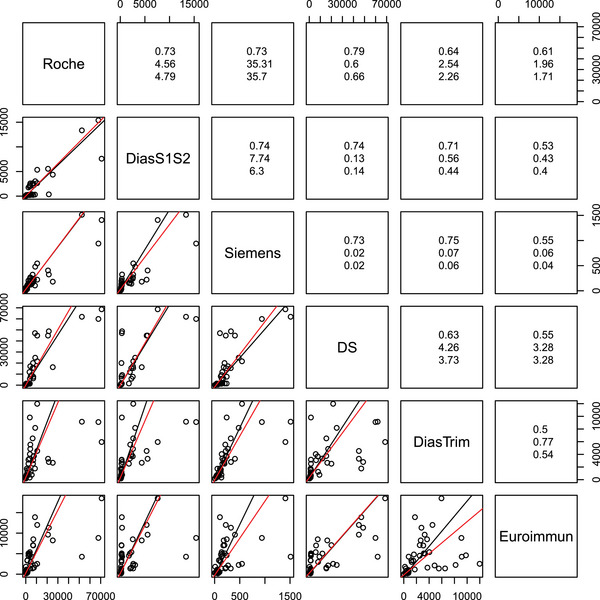
Comparison of mPBR (red lines) to pairwise PBR (black lines) in the SARS‐CoV‐2 test example. In the upper triangle, Kendall's correlation coefficient τ, slope estimates obtained with mPBR (center) and pairwise PBR (bottom) are reported. The unit on all axes is U/mL.

The methodology developed above was applied to find a regression line through data (cf. Ferrari et al. 2021) from 48 samples measured with six different quantitative SARS‐CoV‐2 serological tests^3^ (Figure 3). The estimates for the slopes obtained with the mPBR were compared to the pairwise Passing–Bablok estimates.

The correlation between the different methods is generally quite poor, and even the assumption of a linear relation between some of the measurement pairs may be doubtful. Therefore, linear regression may not be the best way to analyze these data. Despite, or maybe because of these shortcomings, this dataset seems well suited to visualize the differences between the pairwise and multivariate PBR. Moreover, the incompatibility of the pairwise estimates can be considerable, contrasting with the compatibility of the mPBR estimates.

Kendall's correlation coefficient τ ranges from 0.5 between the DiasTrim and the Euroimmun test and 0.79 between the Roche and the DS test. Slope estimators obtained with the multivariate method are mostly less extreme, that is, closer to 1 than the pairwise PBR estimates. While the mPBR estimators are compatible by design, there are considerable incompatibilities between the pairwise PBR estimators. For example, the estimators of DS versus DiasTrim and Euroimmun are 3.73 and 3.28, respectively, from which a DiasTrim versus Euroimmun estimator of 3.28/3.73=0.88 can be calculated, which is more than 60% larger than the one from direct comparison, which is 0.54.

**FIGURE 4:**
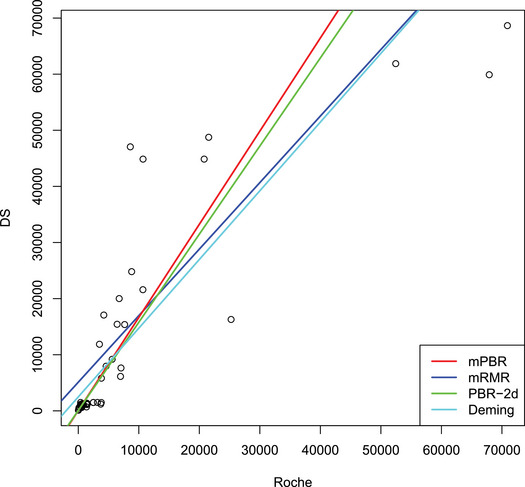
Comparison of the mRMR, Deming, mPBR, and 2D‐PBR fits for the example of the Roche and the DS assay.

A comparison of the Roche and DS assay results, which correlate the most (τ=0.79), shows that the mRMR curve is much more affected by highly influential data points at the upper end of the measurement range than is the mPBR curve (see Figure 4). Both fits coincide much better with their respective 2D‐counterparts. For the other pairwise comparisons, differences between the robust and nonrobust methods are even more pronounced.

**FIGURE 5:**
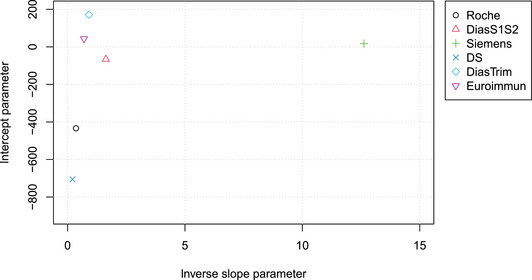
Intercept versus inverse slope parameters of the various SARS‐CoV‐2 serological tests (referring to U/mL). Large differences, especially in b, can be explained by unit U being manufacturer specific.

In Figure 5, the estimated intercept parameters are plotted versus the inverse slope parameters, providing a more condensed representation of the regression estimates. The large differences in slope between some of the assays are due to the use of assay‐specific standards (U/mL), which are different from the international standard established by the WHO, which defined binding antibody units per milliliter (BAU/mL). Therefore, this analysis is rather of the method transformation than the MC type and may be used to calculate conversion factors. For the Roche, Euroimmun, and DS tests, conversion factors of 1 BAU/U were reported by the manufacturers. For the Siemens and Diastrim assay conversion factors of 21.8 BAU/U and 2.6 BAU/U, respectively, were reported. No conversion factor was reported for the DiaS1S2 test. If we neglect the relatively small intercepts, a conversion factor may be estimated for the DIAS1S2 test: Dividing the ${\hat{b}}_{\mu }$ values by the manufacturer‐supplied conversion factors for the five tests for which the factor was available, and taking the geometric mean, yields a mean value $\bar{b}=0.404$ on the WHO BAU/mL scale. Dividing the DiaS1S2 inverse slope parameter $\hat{b}=1.63$ by this mean yields a conversion factor of 4.04 BAU/U for this test.

**FIGURE 6:**
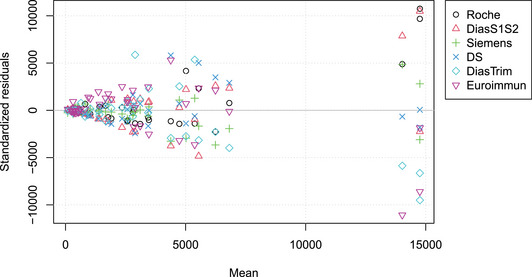
Generalized Bland–Altman plot for the SARS‐CoV‐2 serological tests with $y_{i}-r_{i}-a_{i}$ (standardized residuals) on the ordinate and $r_{i}$ values (mean) on the abscissa.

Finally, in Figure 6, the deviation (“Standardized residuals”) of the $y_{i}$ from the mean ${\hat{r}}_{i}+\hat{a}$, on a common scale, are plotted as a function of ${\hat{r}}_{i}$ (“Mean”), which is similar in spirit to Bland–Altman plots (Bland and Altman 1986). The standard deviations seem to increase linearly with r. For mean values below 10,000, the errors seem to be highest for the Diastrim, DS, and Euroimmun tests, with the DS and Siemens tests showing some systematic bias. At higher values (r>10,000), the Siemens and DS tests show little bias, while the Diastrim and Euroimmun tests appear biased to lower values, and the Roche and DiasS1S2 tests appear biased to higher values.

### 3.2 Bilirubin in Neonates

Thomas et al. (2022) conducted a study comparing total bilirubin measurements in plasma samples from neonates using assays and analysis platforms from different manufacturers.^4^ This article should also be consulted for further details on the assays and the data used below.

**FIGURE 7:**
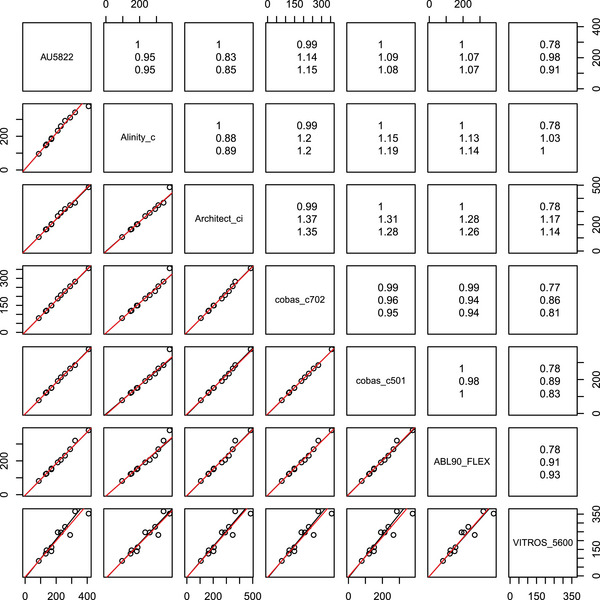
Comparison of 11 neonate total bilirubin plasma samples (concentration in µmol/L) with seven different assays with mPBR (red lines) and pairwise PBR (black lines). In the upper triangle, Kendall's correlation coefficient τ, slope estimates obtained with mPBR (center), and pairwise PBR (bottom) are reported.

While the precision within each method was high, the slopes of the PBR lines differed considerably from 1 for some methods, which could potentially put neonates with high bilirubin plasma levels at risk of not receiving adequate therapy. To address this issue, the data were reanalyzed using mPBR. A total of 11 samples were measured, most of them in duplicates. However, as the second measurement was not available for all systems, only the first measurement was used in the calculations presented below. The mPBR line parameters almost coincide with the ones obtained using pairwise PBR, with only slight differences visible for the comparisons to the VITROS 5600 system, as shown in Figure 7.

**FIGURE 8:**
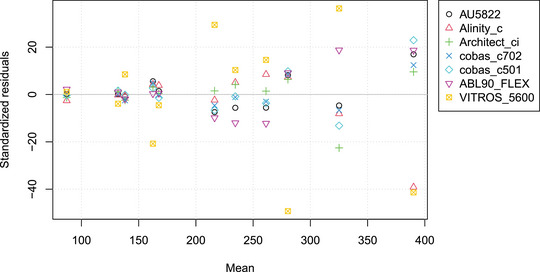
Residuals after scaling versus mean for the 11 bilirubin plasma samples with seven different assays.

**FIGURE 9:**
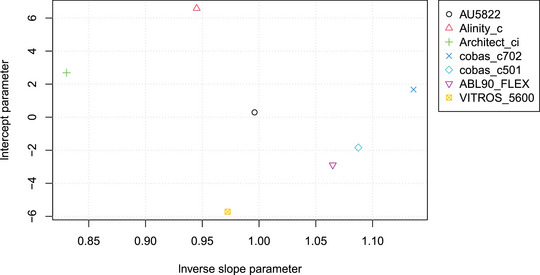
Intercept and inverse slope parameters of the 11 bilirubin plasma samples with seven different assays.

Additionally, the VITROS 5600 system exhibits larger imprecision compared to the other systems (cf. Figure 8), confirming the findings of Thomas et al. (2022). Similar to the previous example, mPBR allows for a condensed representation of the analysis results, as depicted in Figure 9. The figure shows a considerable difference in inverse slope parameters, with the Architect ci 16200 on the lower end and the cobas c702 on the other end.

### 3.3 Comparison of an Assay From Clinical Chemistry

As a third example, the results of a multidimensional MC, involving two different sample preparation methods (A and B), two different measurement platforms (X and Y), and six different reagent lots (1–6) are presented for a quantitative clinical test using 150 whole blood samples. A Bland–Altman type plot of the residuals versus the mean did not show any relevant differences between the assays, so it is not shown.

**FIGURE 10:**
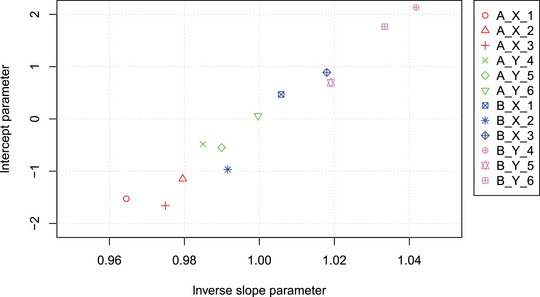
Intercept and inverse slope parameters for the clinical assays differing in sample preparation (A vs. B), instrument platform (X vs. Y), and reagent lot (1–6). Systematic differences between the instrument platform and sample preparation are clearly visible.

However, differences between the two sample preparation methods and, to a lesser extent, between the instrument platforms are clearly visible in the plot of the intercept versus inverse slope parameters, as depicted in Figure 10.

To quantify these differences further, an ordinary least squares (OLS) regression analysis can be performed using the inverse slope parameters and intercept parameters as the response variables, and the relevant effects (preanalytical treatment and instrument platform) as the explanatory variables. Let $x_{\mathrm{pre}}$ be the dichotomous variable representing the preanalytical treatment, with levels 0 for comparator A and 1 for B. Similarly, let $x_{\mathrm{inst}}$ be the dichotomous variable representing the instrument platform, with levels 0 for comparator X and 1 for Y. The linear model

$$-\ln\left(b\right)∼q_{\mathrm{pre}}x_{\mathrm{pre}}+q_{\mathrm{inst}}x_{\mathrm{inst}}$$

is fitted, where $q_{\mathrm{pre}}$ and $q_{\mathrm{inst}}$ are the coefficients describing the linear dependence of −ln(b) on the corresponding x variables. While the intercept has no relevance in this context, ${\hat{\beta }}_{\mathrm{pre}}=\exp\left({\hat{q}}_{\mathrm{pre}}\right)$ and ${\hat{\beta }}_{\mathrm{inst}}=\exp\left({\hat{q}}_{\mathrm{inst}}\right)$ can be interpreted as the average slopes between tests with preanalytical treatment B versus A and instrument platform Y versus X, respectively. For this specific example, ${\hat{\beta }}_{\mathrm{pre}}=0.965$ and ${\hat{\beta }}_{\mathrm{inst}}=0.978$ indicate both a difference in slopes due to preanalytical treatment and a difference due to the instrument platform.

Similarly, the intercept parameters a (without taking logarithms) may be fitted, yielding $\Delta {\hat{a}}_{\mathrm{pre}}=5.55$ and $\Delta {\hat{a}}_{\mathrm{inst}}=3.53$.

This type of analysis requires compatible slope and intercept estimates, which significantly enhances the attractiveness of mPBR compared to pairwise comparisons.

## 4 Discussion

While the comparison of multiple measurement methods is a common task in clinical chemistry, the current methodology used to analyze these comparisons is limited to repeated pairwise comparisons. This approach becomes inefficient, and the resulting reports become more complex as more methods are compared.

In a previous study (Feldmann et al. 1981), a bespoke methodology called mRMR was proposed, but it did not gain much popularity, possibly because even pairwise RMR is not widely used in clinical chemistry. Therefore, the aim of the present article was to extend the well‐established pairwise PBR methodology to the multidimensional setting. The mPBR technique, compared to mRMR, is robust against outlying measurements and also shares the advantages of mRMR, such as the compatibility of slope estimators between pairwise measurement comparisons. The estimators are designed to be equivariant under scaling of the individual measurements, which allows the approach to be applicable not only to MC but also to method transformation. For example, mPBR was applied to the comparison of SARS‐CoV‐2 serological tests (Ferrari et al. 2021), some of which were standardized against different standards, resulting in pairwise slopes vastly different from 1.

When comparing N methods using mPBR, only N−1 parameters need to be estimated, which allows for the calculation of all N(N−1)/2 pairwise slopes and N−1 intercepts. As shown in the Example section, this approach leads to a much more economical representation of the parameters (cf. Figures 5, 9, and 10) and variance structure (Figures 6 and 8) compared to pairwise tabulations and Bland–Altman plots. The inverse slope and intercept parameters, b and a respectively, also allow for further analyses, such as investigating the factors that determine their values.

As an example, in Section 3.3, the influences of preanalytical treatment and instrument platform on the parameters obtained for assays from clinical chemistry were analyzed using OLS. Especially the possibility to compare several method types (like instrument platform, sample preparation, and reagent lot) and whole groups of methods increases the attractiveness of the regression method considerably.

As far as the mathematical formulation of the mPBR estimator is concerned (cf. Section 2.5), it relates to the field of directional statistics (Fisher, Lunn, and Davies 1993; Mardia, Jupp, and Mardia 2000), which opens up interesting possibilities for further exploration. A proof of the consistency of the mPBR estimator is given in the appendix, and robustness could at least be shown in the limit that the measurements which are not outliers are very precise. Generally, it can be stated that the theoretical basis of MC is by far not as developed as that of ordinary, even nonlinear, regression and brings with it subtle new problems.

In the analysis of MC study data, simply estimating a regression line is not sufficient. Confidence intervals for the slope, intercept, and bias at medical decision points should also be reported. The most common technique for estimating confidence intervals for regression, including PBR, is bootstrapping (Carpenter and Bithell 2000). This technique involves repeatedly estimating point estimates on random samples from the original dataset. Bootstrapping can also be applied to mPBR. Once confidence intervals have been determined, questions about the significance of deviations from the slope of 1 or the intercept of 0 can be answered.

Regarding the practical implementation of the proposed algorithm, at the moment, only a basic variant of the Weiszfeld algorithm has been implemented. This algorithm scales at least quadratically with the number of observations and requires all samples to have been measured with all methods. In the future, it might be possible to impute missing values using an expectation maximization type of algorithm.

A further topic for future studies may be an extension of robust methodology to estimate nonlinear relations between the measurements. Nonrobust polynomial regression methods have been discussed (Cheng and Van Ness 1999) but lead to rather complicated equations. More pragmatic approaches (Hawkins and Sharma 2010), based on estimating a hierarchy of multiplicative interaction terms, seem to be promising especially in the field of clinical chemistry.

## Conflicts of Interest

Florian Dufey is an employee of Roche Diagnostics GmbH and holds shares in F Hoffman‐La Roche Ltd.

## Open Research Badges

This article has earned an Open Data badge for making publicly available the digitally‐shareable data necessary to reproduce the reported results. The data is available in the Supporting Information section.

This article has earned an open data badge “**Reproducible Research**” for making publicly available the code necessary to reproduce the reported results. The results reported in this article could fully be reproduced.

## Supporting information

Supporting Information

## Figures and Tables

**FIGURE A1:**
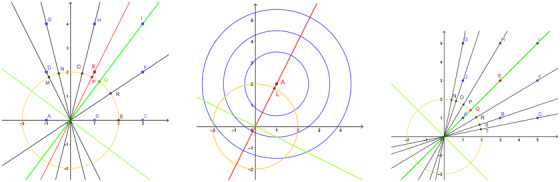
Left figure: The estimator from Equation (8) will in general be inconsistent, even in 2D and for symmetric error distributions of the iid components. Central figure: The same estimator is consistent if the errors are normal. Right figure: With the mPBR equivariance assumption $\Lambda =\mathrm{diag}(b^{2})$, consistent estimators will be obtained for general error distributions of the iid components.

**FIGURE B1:**
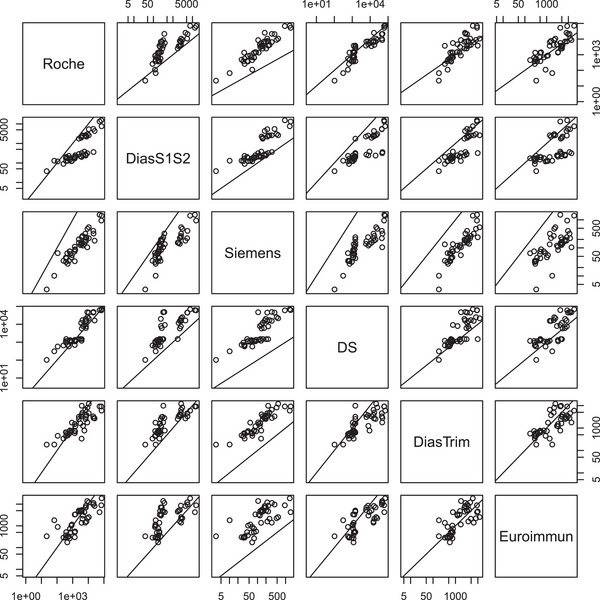
Pairwise plot of the SARS data from the different tests on a log–log scale. Lines are diagonal x=y.

## Data Availability

Data used in Section 3.1 are available as supplementary material from the article by Ferrari et al. (2021): https://ars.els‐cdn.com/content/image/1‐s2.0‐S0009898121003028‐mmc1.docx. Data used in Section 3.2 are taken from the article by Thomas et al. (2022). All data, including the ones used in Section 3.3, are available together with the code to reproduce the analyses as an R‐package as supplementary material.

## Endnotes

1 A direct minimization of the sum of squares with this constraint of Λ, and Ξ would yield inconsistent results (cf. Pronzato and Pázman 2013).

2 For example, let the unit vector of the point O be q and take the coordinates of the outliers as $x_{i}=\gamma iq$. If the coordinates of the correct points are fixed, in the limit γ→∞, all pairwise slopes involving at least one outlier will get projected on the point q

3 Full names of the tests and abbreviations used in the text and captions: Diasorin LIAISON SARS‐CoV‐2 Trimerics IgG (DiasTrim), Euroimmun Anti‐SARS‐CoV‐2 (Euroimmun), Roche Elecsys Anti‐SARS‐CoV‐2‐S (Roche), Diesse CHORUS SARS‐CoV‐2 “NEUTRALIZING” Ab (DS), Atellica IM SARS‐CoV‐2 IgG (Siemens), Diasorin LIAISON SARS‐CoV‐2 S1/S2 IgG (DiaS1S2).

4 Names of the analysis platforms and manufacturers: Alinity c and Architect ci16200 (Abbott Diagnostics, USA), AU5822 (Beckman Coulter, USA), cobas c702 and cobas c502 (Roche Diagnostics, Germany), and ABL90 FLEX (Radiometer Denmark).

## Appendix A Consistency of the Slope Estimator

Without the restriction of generality, we assume that the $x_{i}$ is scaled such that $\Lambda =I_{N}$ so that the $\varepsilon_{i\mu }$ for fixed i are iid. (a) The estimator obtained from Equation (8) is consistent if all errors $\varepsilon_{i\mu }$ for fixed i are iid normal; Mere symmetry $\mathrm{pdf}\left(\varepsilon_{i\mu }\right)=\mathrm{pdf}\left(-\varepsilon_{i\mu }\right)$ of the error distributions is not sufficient. (b) The mPBR estimator with 0 intercept (Equation 9) is consistent, given the mPBR assumption $E(x_{i})∼e$. (c) Also, the mPBR estimator with nonzero intercept is consistent, given the mPBR assumption $E(\Delta x_{ij})∼e$.

(a) Let ${\hat{\theta }}_{n}$ be an extremum estimator with
  $${\hat{\theta }}_{n}=\underset{\theta \in \Theta }{\arg\max}\;{\hat{Q}}_{n}\left(\theta \right)$$
  and $\theta_{0}$ being the true value. If (Newey and McFadden 1994, Theorem 2.1) there exists a function $Q_{0}\left(\theta \right)$ such that
  (i) $Q_{0}\left(\theta \right)$ is uniquely maximized at $\theta_{0}$;
  (ii) Θ is compact;
  (iii) $Q_{0}\left(\theta \right)$ is continuous;
  (iv) ${\hat{Q}}_{n}\left(\theta \right)$ converges uniformly in probability to $Q_{0}\left(\theta \right)$,
  then the estimator ${\hat{\theta }}_{n}$ is consistent, that is ${\hat{\theta }}_{n}\overset{P}{\to }\theta_{0}$.
  We consider the sequence of estimates when there is a discrete finite number of concentrations and each concentration gets measured n times with n→∞. Then it is sufficient to show convergence for the repeated measurement of a single concentration r so that all $r_{i}=r=\mathrm{const}.$, with iid errors $\varepsilon_{i\mu }$. Let $\theta =\tilde{s}$.
  (ii) |θ|=1, hence Θ is compact.
  (iv) The $f\left(x_{i},\theta \right)=\arccos\left(\left|{\tilde{s}}^{T}{\tilde{x}}_{i}\right|\right)$ are then also iid, and $\hat{Q}\left(\theta \right):=-\sum_{i=1}^{n}f\left(x_{i},\theta \right)/n$ converges in probability to $Q_{0}\left(\theta \right):=-E_{\theta_{0}}\left(f\left(x,\theta \right)\right)$ by the law of large numbers. As f is bounded and θ is compact, this convergence is uniform in θ.
  (iii) As f(x,θ) is continuous, $Q_{0}\left(\theta \right)$ will be continuous if the distribution of ε is continuous.
  (i) Even in two dimensions and for symmetric error distributions, the function $Q_{0}\left(\theta \right)$ is in general not maximized at $\theta_{0}$. A counterexample is shown in the left Figure A1, where E(x)=(1,2), $\theta_{0}=\arctan2,$ and the distribution of the components of ε is strongly localized near {−1,0,1} with equal weights. The maximum of $Q_{0}\left(\theta \right)$ is at $\hat{\theta }=\arctan\left(4/3\right)$. Similar counterexamples can be constructed in higher dimensions.
  A sufficient condition for the function $Q_{0}\left(\theta \right)$ to be maximized at $\theta =\theta_{0}$ is a rotationally symmetric distribution of ε (cf. the central Figure A1). In this situation, the direction of E(x), $\theta_{0}$, is the only distinguished direction; hence, the maximum has to coincide with it. However, the only rotationally symmetric distribution with iid components is a multidimensional Gaussian (Kac 1939).
(b) In the case of the mPBR estimator with zero intercept (Equation 9), the scaling vector b may be restricted to |b|=1, then the left‐hand side of this equation, $f(x_{i},\theta ,b)$, fulfills (ii)–(iv).
  Then for the estimator to be consistent the expected value
  $$Q_{0}\left(\tilde{s},b\right)=-E\left(\arccos\left(\left|{\tilde{s}}^{T}\tilde{Xb}\right|\right)\right)$$
  must be maximal for $\tilde{s}=e$ and b=e. A necessary condition is that its derivative with respect to $\tilde{s}$ vanishes,
  $$E\left(\mathrm{sgn}\left(x^{T}e\right)\frac{Px}{\left|Px\right|}\right)=0.$$
  The proof of this condition rests on the distribution of x being invariant under permutations π of the components $\left\{x_{\mu }\right\}$; call $x_{\pi }$ the result of such a permutation (cf. right Figure A1). With the region $R=-\infty <x_{1}\leq x_{2}\leq \cdots \leq x_{N}<\infty$ being a stabilizer of the permutation group $S_{N}$, this expectation value can be written as
  $$\int_{x\in R}d^{N}x\prod_{\mu =1}^{N}p{\left(x_{\mu }\right)}^{N}\sum_{\pi }\mathrm{sgn}\left(x_{\pi }^{T}e\right)\frac{Px_{\pi }}{\left|Px_{\pi }\right|}$$
  Now, $x_{\pi }^{T}e=x^{T}e$ and $|Px_{\pi }\left|=|Px\right|$ for all permutations π which can therefore be pulled out of the sum. But^5^ $\sum_{\pi }x_{\pi }=\left(N-1\right)!\sqrt{N}\sum_{\mu }x_{\mu }e$ so that $P\sum_{\pi }x_{\pi }=0$.
(c) It remains to extend these proofs for the mPBR with nonvanishing intercepts. To this end, it is sufficient to consider the estimators arising from repeated measurements of two samples which leads to three contributions, namely the distribution of the differences of repeated measurements of sample 1 and sample 2, $\Delta x_{11}$ and $\Delta x_{22}$, respectively, and the distribution of the repeated distances between samples 1 and 2, $\Delta x_{12}$. In analogy to the situation without intercept, for all three terms, the average angular distances will be minimal from the space diagonal e.□

We considered here only the simplest case, where with increasing n, individual samples are measured repeatedly. It is possible to extend the proof to the more interesting situation where each sample is measured only once but the number of samples increases instead. The proof given will hold also in this situation provided the limiting distribution of the $x_{i}$ exists and the distributions of its components $x_{\mu }$ are identical.

## Appendix B Plot of the SARS Data on a Log–Log Scale

Most of the SARS‐CoV 2 data discussed in Section 3.1 cluster at small values, which makes it difficult to assess the correlation of the data from Figure 3 alone. Therefore, in Figure B1, a plot with both axes being logarithmized is shown. Both grouping and deviations from the assumed linear behavior can be identified.
