# Supplementary figures and images for: Robust Regression Techniques for Multiple Method Comparison and Transformation
Source: Biom J. 2024 Jul 13;66(5):e202400027. doi: 10.1002/bimj.202400027 (PMC12859536; doi:10.1002/bimj.202400027)

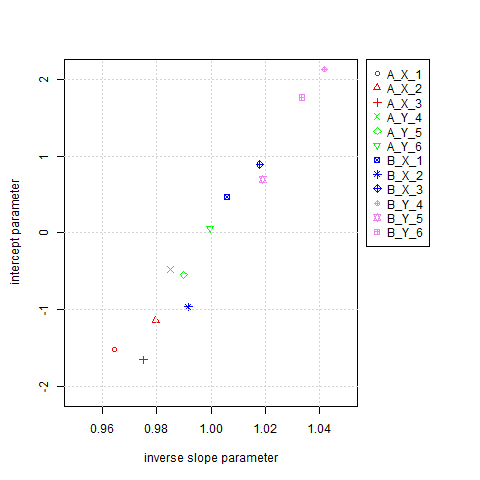

Supplement: Supplementary file 1 — Supporting Information [file BIMJ-66-e202400027-s001.zip › results/figure10.png]

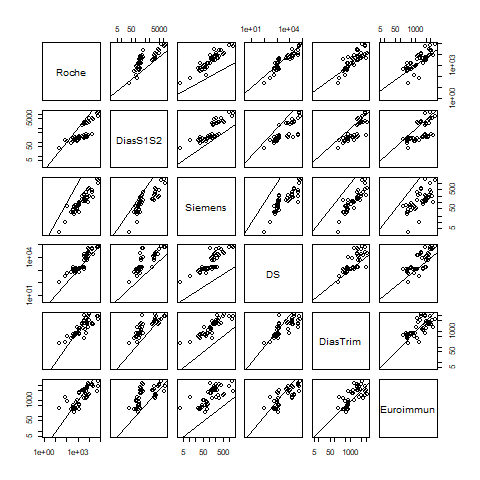

Supplement: Supplementary file 1 — Supporting Information [file BIMJ-66-e202400027-s001.zip › results/figure12.png]

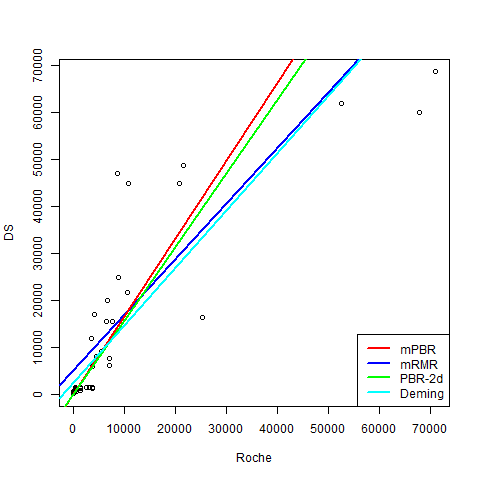

Supplement: Supplementary file 1 — Supporting Information [file BIMJ-66-e202400027-s001.zip › results/figure3.png]

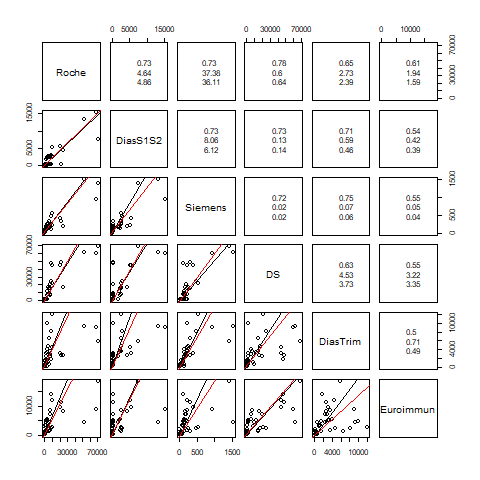

Supplement: Supplementary file 1 — Supporting Information [file BIMJ-66-e202400027-s001.zip › results/figure4.png]

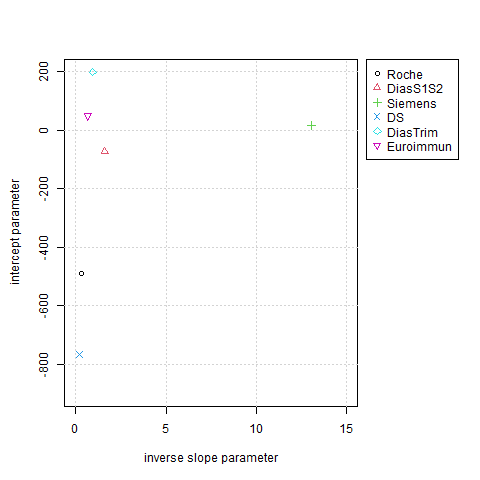

Supplement: Supplementary file 1 — Supporting Information [file BIMJ-66-e202400027-s001.zip › results/figure5.png]

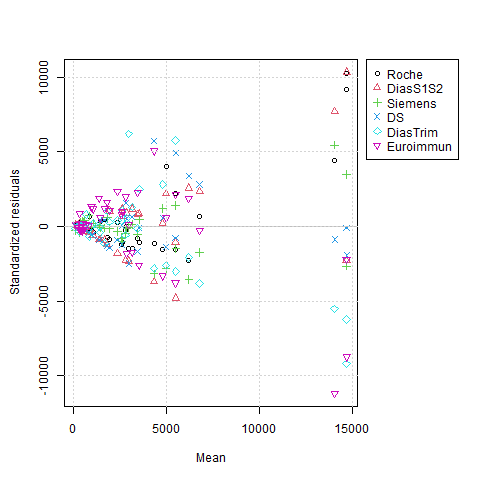

Supplement: Supplementary file 1 — Supporting Information [file BIMJ-66-e202400027-s001.zip › results/figure6.png]

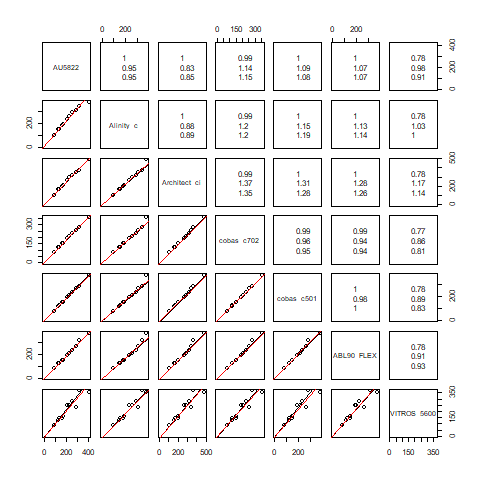

Supplement: Supplementary file 1 — Supporting Information [file BIMJ-66-e202400027-s001.zip › results/figure7.png]

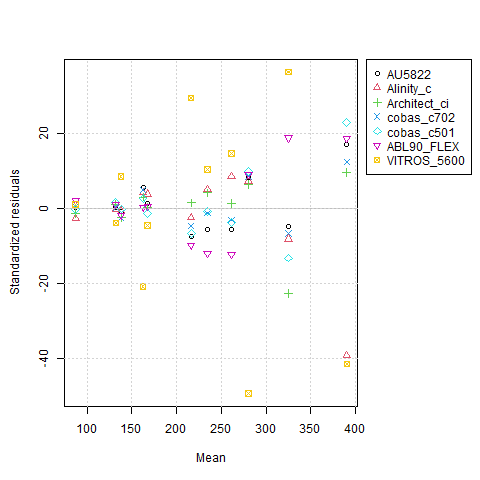

Supplement: Supplementary file 1 — Supporting Information [file BIMJ-66-e202400027-s001.zip › results/figure8.png]

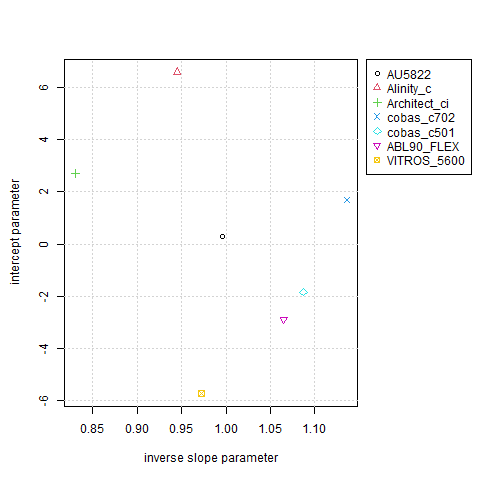

Supplement: Supplementary file 1 — Supporting Information [file BIMJ-66-e202400027-s001.zip › results/figure9.png]
